# Higher Redox State of Coenzyme Q10 Is Associated with Higher Risk of All-Cause Mortality in a Sample from the Northern German General Population

**DOI:** 10.3390/antiox15030343

**Published:** 2026-03-09

**Authors:** Paula Stürmer, Katharina S. Weber, Eike A. Strathmann, Cara Övermöhle, Jakob C. Voran, Frank Döring, Matthias Laudes, Wolfgang Lieb

**Affiliations:** 1 Institute of Epidemiology, Kiel University, 24105 Kiel, Germany; 2 Department of Internal Medicine III (Cardiology and Intensive Care), University Hospital Schleswig-Holstein, 24105 Kiel, Germany; 3 DZHK (German Centre for Cardiovascular Research), Partner Site Hamburg/Kiel/Lübeck, 10785 Berlin, Germany; 4 Division of Molecular Prevention, Institute of Human Nutrition and Food Science, Kiel University, 24118 Kiel, Germany; 5 Institute of Diabetes and Clinical Metabolic Research, University Hospital Schleswig-Holstein, 24105 Kiel, Germany

**Keywords:** Coenzyme Q10, ubiquinol, ubiquinone, Coenzyme Q10 redox state, all-cause mortality, general population

## Abstract

Coenzyme Q10 (CoQ10) plays an important role in human health; for example, through the antioxidant function of its reduced form (ubiquinol). As the long-term health effects of circulating CoQ10 remain largely unknown, we examined the association of total CoQ10, ubiquinol, ubiquinone (oxidized CoQ10), and CoQ10 redox state (percentage of ubiquinone in total CoQ10) with all-cause mortality in a sample from the northern German general population. In *n* = 1333 individuals (60.1% females, median baseline age: 48.0 years [37.7; 58.0]), serum total CoQ10, ubiquinol, ubiquinone, and CoQ10 redox state were measured at baseline and found to be related to all-cause mortality using Cox regression models (adjusted for sex, age, body mass index, smoking, systolic blood pressure, total cholesterol, diabetes, and C-reactive protein). After 12.9 years [12.4; 17.1], *n* = 123 deaths had occurred. A higher CoQ10 redox state was independently associated with a higher risk of all-cause mortality after multivariable adjustment (HR: 1.18 [95% CI 1.02–1.36] per 1-SD increment, HR: 1.92 [95% CI 1.16–3.17] for tertile 3 vs. tertile 1), while higher ubiquinone levels were associated with greater all-cause mortality risk only in the unadjusted model. A higher CoQ10 redox state was associated with a higher risk of all-cause mortality in a population-based sample, possibly indicating detrimental long-term health effects of the lower antioxidant capacity of CoQ10.

## 1. Introduction

Coenzyme Q10 (CoQ10) is an endogenously synthesized lipid-soluble molecule with important implications for human health that is present in all intracellular membranes [1]. Amongst other functions, CoQ10 is vital for ATP derivation as it is a coenzyme in the electron transport chain [2], and it plays a role in the modulation of gene expression and mitochondrial function and signaling [3]. Furthermore, anti-inflammatory properties of CoQ10 have been described. For example, CoQ10 supplementation significantly decreased the levels of inflammatory markers, such as C-reactive protein (CRP) [4,5]. Most importantly, ubiquinol, the reduced form of CoQ10, constitutes an effective antioxidant in that it interferes with lipid, protein, and DNA oxidation in the cells. Also, in circulation, where CoQ10 is bound to lipoproteins, ubiquinol can protect the lipoproteins from oxidative damage [1], a mechanism by which ubiquinol may mitigate endothelial damage and thus contribute to the prevention of cardiovascular diseases (CVDs) [6]. In addition to its direct antioxidant effects, ubiquinol can reduce radicals of other antioxidative agents, such as α-tocopherol [2,6].

Taken together, CoQ10 is important for cardiovascular health, mainly due to its role in energy metabolism, its capacity to counteract the oxidation of low-density lipoprotein (LDL)-cholesterol, and its protective effect on endothelial function [7]. Consequently, low CoQ10 status has been associated with various diseases, including hypertension, diabetes mellitus, chronic kidney disease, and CVD, for which it has also been proposed as a potential prognostic biomarker [8,9]. In heart failure patients, for example, CoQ10 deficiency was associated with an increased risk of mortality [10]. On a parallel note, evidence has accumulated that CoQ10 supplementation could reduce hospitalization and mortality risk in this patient group [11,12].

Despite mounting evidence regarding the detrimental health effects of low CoQ10 status, as well as the likely effectiveness of CoQ10 supplementation for certain diseases, little is known about the association between CoQ10 and survival in the community. In a sample from the general population in the United States, intake of oral CoQ10 supplementation was not associated with the risk of all-cause or cardiovascular mortality [13]. By contrast, a four-year intervention with oral supplements containing CoQ10 and selenium significantly reduced the risk of cardiovascular mortality in healthy elderly individuals [14,15]. However, to the best of our knowledge, the association between circulating CoQ10 and mortality risk in individuals from a community has not been systematically investigated thus far. Therefore, we aimed to assess the association of different biomarkers of CoQ10 status with all-cause mortality risk in a community-based sample. Specifically, we investigated the serum concentrations of total CoQ10, ubiquinol, and ubiquinone (oxidized CoQ10) and the redox state of CoQ10, which describes the percentage of ubiquinone in total CoQ10, and related each biomarker individually to long-term survival in a sample from the northern German general population.

## 2. Materials and Methods

### 2.1. Study Sample

The study sample for the present prospective analyses consists of three subsamples from the same region (city of Kiel) in northern Germany: a subsample of the popgen control cohort of the popgen Biobank, Kiel, and two subsamples of the Food Chain Plus (FoCus) cohort, which are described in detail in [16,17], respectively. The subsample of the popgen control cohort (subsample A) consists of *n* = 569 healthy blood donors, who were recruited from the University Hospital in Kiel in 2007. For FoCus, between 2011 and 2014, a reference sample of *n* = 1305 individuals were randomly recruited via population registries in Kiel (subsample B), while *n* = 501 individuals with obesity (defined as body mass index (BMI) > 30 kg/m^2^; subsample C) were recruited from the Obesity Outpatient Clinic of the University Hospital in Kiel. All relevant clinical and physiological data (used as covariates in our statistical analyses) and the biosamples for the analyses of CoQ10 biomarkers were obtained from the respective baseline examinations (in 2007 for subsample A; between 2011 and 2014 for subsamples B and C). Importantly, across these subsamples, the clinical and physiological data were ascertained in a comparable manner. The outcome data on all-cause mortality was ascertained after a median follow-up of 12.9 years [12.4; 17.1]. After excluding individuals with missing information on CoQ10 (*n* = 997), BMI (*n* = 3), or smoking habits (*n* = 2) and those lost to follow-up (*n* = 40), the analytical sample comprised *n* = 1333 individuals (Figure S1). Both the popgen control cohort and the FoCus cohort were approved by the ethical review board of the Medical Faculty of Kiel University (project identification code A 156/03) and are consistent with the Declaration of Helsinki. All individuals provided written informed consent.

### 2.2. Ascertainment of Vital Status

Vital status of the participants was ascertained in 2024 (subsample A) and 2025 (subsamples B and C) via population registries. As no information on cause of death was available, we only considered all-cause mortality for our analyses.

### 2.3. Covariate Assessment

Covariate assessment was performed during baseline examinations (in 2007 for subsample A; between 2011 and 2014 for subsamples B and C). For all subsamples, trained personnel performed in-house examinations at the University Hospital in Kiel. Blood pressure was measured in a sitting position by use of a sphygmomanometer after an initial resting phase of at least 5 min. For subsample A, one measurement was performed. For subsamples B and C, blood pressure measurement was performed in duplicate with a 3 min interval between measurements. The arithmetic mean of both measurements was considered for analyses. BMI was calculated as weight [kg] divided by height squared [m^2^]. Furthermore, blood samples were drawn in a sitting position. For serum samples, serum separator tubes (Sarstedt AG, Nümbrecht, Germany) were used, and the samples were centrifuged, aliquoted, and stored at −80 °C until further analysis. In unfrozen EDTA plasma samples on the day of blood collection, CRP was measured applying immunoturbidimetry, while total cholesterol and glucose were measured photometrically [17]. Diabetes was defined based on plasma glucose levels (≥126 mg/dL in a fasted state; ≥200 mg/dL in a non-fasted state) and on intake of antidiabetic medication. Information on smoking habits (never smoked or current smokers) was ascertained through standardized questionnaires.

### 2.4. Coenzyme Q10 Measurement

For the present analyses, CoQ10 biomarkers were assessed in serum samples that were obtained during the baseline examinations and subsequently stored at −80 °C. Specifically, serum ubiquinol-10 and ubiquinone-10 were measured in 2011–2012 for subsample A and in 2013–2014 for subsamples B and C (i.e., after a maximum storage time of 5 years), as described in detail elsewhere [18,19].

In brief, internal standards (56 pmol ubiquinol-9 and 9 pmol ubiquinone-9 dissolved in 50 µL ethanol) were added to a 50 µL serum aliquot, which was subsequently subjected to hexane extraction (500 µL) and centrifugation (5 min, 1000× *g*, 4 °C). Further, the hexane phase was evaporated under a stream of argon, and the dry residue was re-dissolved in ethanol (50 µL) for injection into the high-pressure liquid chromatography system (Model 2250 pump, Bischoff, Leonberg, Germany; Rheodyne injection valve, Rheodyne, Cotati, CA, USA; Prontosil 120-3-C18-SH PEEK column, Bischoff, Leonberg, Germany) with electrochemical detection (Coulochem II electrochemical detector; ESA, Bedford, MA, USA).

Subsequently, total CoQ10 was calculated as the sum of ubiquinol and ubiquinone. CoQ10 redox state was calculated as the percentage of ubiquinone in total CoQ10.

### 2.5. Statistical Analyses

Categorical variables are reported as absolute numbers and percentages and continuous variables are reported as medians [quartile 1; quartile 3]. We performed all analyses using RStudio (v2024.12.0, R for windows 4.3.1). Statistical significance was considered at *p* < 0.05.

For survival analyses, we considered total CoQ10, ubiquinol, ubiquinone, and CoQ10 redox state each as separate exposure variables and all-cause mortality as the outcome variable. The exposure variables were modeled both as continuous (effect estimates per 1-SD increment) and as categorical traits (divided into tertiles, with the bottom tertile as a reference). We applied Kaplan-Meier Curves and Log-rank tests to graphically display the unadjusted association between tertiles of the exposure variables with survival duration. Cox proportional hazard regression models were used to quantify these associations and to adjust for relevant confounders. Time alive between the baseline examination and the date of death or the date of censoring was considered the underlying time variable. The proportional hazard assumption was tested using the Schoenfeld residual method, and no violation was noted. We report an unadjusted model (Model 1), a minimally adjusted model including sex [male/female] and age [years] as covariates (Model 2), and a fully adjusted model further including BMI [kg/m^2^], smoking habit, [ever/never], systolic blood pressure [mmHg], diabetes prevalence [yes/no], and total cholesterol [mmol/L] (Model 3). Additionally, as a previous analysis by Fischer et al. (2016) in our study sample showed a direct correlation between CoQ10 redox state and CRP [20], a Model 4 including all covariates of Model 3 plus CRP was applied. To test for non-linear associations between exposure variables and all-cause mortality, we performed restricted cubic spline analyses in Model 4 with knots placed at the 10th, 50th and 90th percentiles using the R package *plotRCS* [21]. Further, as subsamples differed by baseline characteristics, including BMI, we tested for effect modification by subsample and BMI.

Additionally, we performed two sensitivity analyses. First, as the CoQ10 redox state is dependent on total CoQ10 levels, we included total CoQ10 as a covariate in the fully adjusted Cox regression models, with CoQ10 redox state as the exposure. Second, to rule out reverse causality, survival analyses were repeated after exclusion of individuals who died within two years after the baseline examination.

## 3. Results

### 3.1. Characterization of the Study Sample

The analytical sample comprised *n* = 1333 individuals with a baseline median age of 48.0 years [37.7; 58.0] and was 60.1 % female. Characteristics of the overall study sample are presented in Table 1. Over the course of 12.9 years [12.4; 17.1] of follow-up, a total of *n* = 123 individuals died. Serum CoQ10 levels at baseline were within a normal physiological range [22], with a median ubiquinol of 0.71 µmol/L [0.55; 0.89]. Total CoQ10 showed an interquartile range from 0.64 to 1.03 µmol/L and ubiquinone from 0.08 to 0.13 µmol/L, leading to a redox state of CoQ10 of 13.0 % [11.8; 14.4]. Individuals who died during the follow-up period tended to have higher baseline CoQ10 levels, a higher CoQ10 redox state, higher CRP values, were older, more likely to be male, and had a higher BMI than individuals still alive at the end of the study period. Characteristics of the study participants according to the three subsamples of the study sample are presented in Table S1. As expected, sample characteristics differed for some variables (e.g., BMI and age) due to different recruitment strategies (blood donors vs. a sample from population registries vs. patients from an obesity outpatient clinic). Variables with relevant differences between subsamples were included as covariates in our statistical analyses and effect modification by subsample was tested.

### 3.2. Association of Coenzyme Q10 with All-Cause Mortality

**Figure 1:**
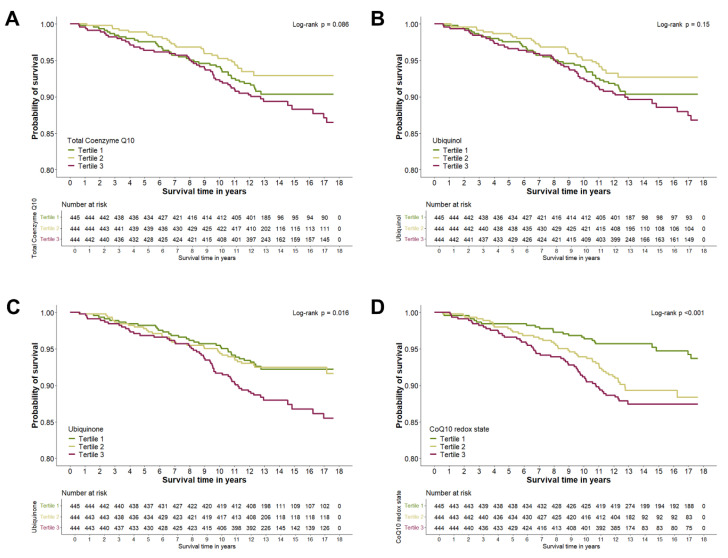
Kaplan–Meier Curves displaying the unadjusted association of total Coenzyme Q10 (**A**), ubiquinol (**B**), ubiquinone (**C**), and CoQ10 redox state (**D**) with survival duration in *n* = 1333 individuals. Abbreviations: CoQ10, Coenzyme Q10.

**Figure 2:**
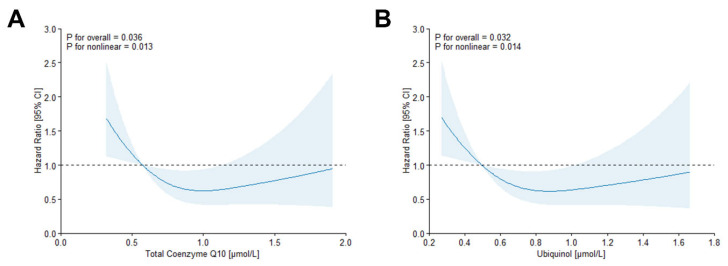
Restricted cubic spline analysis of total Coenzyme Q10 (**A**) and Ubiquinol (**B**) with all-cause mortality, with knots placed at the 10th, 50th, and 90th percentiles. Analyses adjusted for sex, age, body mass index, systolic blood pressure, smoking habits, diabetes prevalence, total cholesterol, and C-reactive protein.

In unadjusted analyses illustrated by Kaplan-Meier Curves (Figure 1), Log-rank tests indicated associations between survival probability and ubiquinone (p_Log-rank_ = 0.016) and CoQ10 redox state (p_Log-rank_ < 0.001), respectively, while no clear association could be observed for total CoQ10 (p_Log-rank_ = 0.086) or ubiquinol (p_Log-rank_ = 0.15) with survival probability. In Cox regression models (Table 2), the higher all-cause mortality risk associated with higher ubiquinone observed in the unadjusted model was rendered statistically non-significant after multivariable adjustment. By contrast, a higher all-cause mortality risk was observed with a higher CoQ10 redox state in all models applied (Model 4: Hazard Ratio (HR): 1.18 [95% Confidence Interval (CI) 1.02–1.36] per 1-SD increment, HR: 1.92 [95% CI 1.16–3.17] for tertile 3 vs. tertile 1). In restricted cubic spline analyses, both total CoQ10 (Model 4: p_overall_ = 0.036, p_non-linear_ = 0.013) and ubiquinone (Model 4: p_overall_ = 0.032, p_non-linear_ = 0.014) were statistically significantly overall and non-linearly associated with all-cause mortality risk (Figure 2). Here, a lower all-cause mortality risk was observed with increasing levels of both biomarkers, yet these associations reached statistical significance only in mid-range levels. Accordingly, Cox regression models showed a lower all-cause mortality risk when comparing tertile 2 to tertile 1 (Model 4: HR_total CoQ10_: 0.50 [95% CI 0.31–0.82], HR_ubiquinol_: 0.55 [95% CI 0.34–0.90]), while statistical significance was not reached when comparing tertile 3 to tertile 1 or when considering the exposures as continuous traits. No effect modification by subsample or BMI (p_interaction_ > 0.05, respectively) was apparent for any of the exposures.

### 3.3. Sensitivity Analyses

Both the inclusion of total CoQ10 as a covariate when modelling CoQ10 redox state as the exposure (Table 2) and the exclusion of individuals who died within two years after baseline (*n* = 8) to rule out reverse causality (Table S2) resulted in virtually no changes in the results of the main analysis.

## 4. Discussion

We related four markers of CoQ10 status to the risk of all-cause mortality in a moderate-sized population-based sample (*n* = 1333) from the north of Germany (median follow-up time, 12.9 years; *n* = 123 deaths). Our principal observations were as follows: First, we observed a higher CoQ10 redox state to confer a statistically significant higher risk of all-cause mortality, which held true after adjusting for well-established risk factors and after correcting for total CoQ10 concentrations. Second, higher levels of ubiquinone showed a trend towards an association with higher all-cause mortality risk, which reached statistical significance only in the unadjusted model. Third, for total CoQ10 and ubiquinol, we observed a statistically significant overall and non-linear association with all-cause mortality, respectively.

### 4.1. In the Context of the Published Literature

CoQ10 has numerous important functions in the human body and is therefore tightly linked to health and disease. Several lines of evidence suggest an important role of CoQ10 in aging and age-related diseases, such as CVD, probably through its role in maintaining mitochondrial function and its antioxidant and anti-inflammatory effects [6,8]. In patients who experienced cardiac arrest, CoQ10 levels were lower than in healthy controls and were a predictor of in-hospital mortality [23]. Likewise, in patients with chronic heart failure, lower plasma CoQ10 was associated with an increased risk of mortality [10]. By contrast, in patients with the same condition, supplementation with CoQ10 increased blood levels of CoQ10 and improved cardiac function while reducing mortality and hospitalization risk [11,12]. Also, in healthy individuals, supplementation of CoQ10 combined with selenium for four years significantly reduced the risk of CVD mortality [14], while self-reported supplementation of CoQ10 was not associated with mortality risk in a sample from the US adult population [13]. To the best of our knowledge, we are the first to report the association between circulating CoQ10 and the risk of all-cause mortality in a sample from the general population without focusing on CoQ10 supplementation.

The reduced form of CoQ10, ubiquinol, is an endogenously synthesized antioxidant involved in numerous redox processes in the organism. It protects lipids, proteins, and DNA from oxidative damage and regenerates other main antioxidants, such as α-tocopherol [1,24]. The oxidized form of CoQ10, ubiquinone, which is generated in these antioxidant processes, is reduced to ubiquinol by reductases that transfer electrons from NAD(P)H in the cytosol, maintaining the antioxidant capacity of CoQ10 [24]. Consequently, the CoQ10 redox state, meaning the percentage of ubiquinone in total CoQ10, is considered a marker of oxidative stress [25]. One of the key functions of CoQ10 in the circulation is to protect LDL-cholesterol from oxidative damage [26]. By this mechanism, CoQ10 may protect against vascular damage and CVD since oxidized LDL-cholesterol constitutes a strong predictor of cardiovascular risk [27]. Consequently, oxidative damage and stress play important roles in the development of various chronic diseases, such as CVDs [28], which rank among the highest leading causes of death worldwide [29]. In our analyses, we observed a higher all-cause mortality risk in individuals with a higher CoQ10 redox state in the serum, possibly indicating a lower capacity of CoQ10 to protect lipoproteins such as LDL-cholesterol from antioxidative damage. In line with our observation, in a Swedish intervention study, four years of supplementation with CoQ10 and selenium improved the systemic redox status and was indicative of reduction in the risk of CVD mortality [30]. Furthermore, a decrease in total thiol levels, which constitute a proxy for the redox control status in the circulation, was strongly associated with higher risk of all-cause and CVD mortality in European cohorts [31]. Hence, the association between a higher CoQ10 redox state and the higher all-cause mortality risk observed in our analyses might, at least in part, be attributable to a lower antioxidant capacity of CoQ10 in the circulation, possibly impacting CVD occurrence and severity. However, as no information on cause of death was available in our analyses, the latter remains speculative and should be addressed in future studies.

In general, we observed higher levels of total CoQ10, ubiquinol, and ubiquinone, as well as a higher redox state, in individuals who died than in individuals still alive at the end of the study period. In comparison, higher total CoQ10 and ubiquinol levels, as well as a higher ratio of ubiquinol to ubiquinone, were observed in individuals with metabolic syndrome compared to healthy individuals, with the authors concluding that this might indicate an adaptive response to oxidative stress in metabolic syndrome [32]. In our analyses, in contrast to the CoQ10 redox state, the higher mortality risk observed with higher ubiquinone levels did not hold true after multivariable adjustment. Similarly, higher total CoQ10 and ubiquinol showed a trend towards an inverse association with mortality risk but did not reach statistical significance. These observations might indicate that either (A), our study was simply not sufficiently powered to detect smaller effect sizes (80% power was reached for our analyses at HRs of 0.60 or 1.66 in the main analysis; HRs for total CoQ10, ubiquinol and ubiquinone were considerably smaller) or (B) that the redox state, meaning the percentage of ubiquinone in total CoQ10, constitutes the more informative marker when relating markers of CoQ10 to health outcomes compared to ubiquinol or ubiquinone alone.

In addition to its antioxidant effects, CoQ10 is also ascribed anti-inflammatory properties. In supplementation studies, CoQ10 supplements could effectively reduce levels of proinflammatory biomarkers, such as CRP, possibly by inhibiting NF-κB gene expression [4,5]. In a previous analysis by Fischer et al. (2016) in our study population, higher CRP concentrations were observed in individuals with a higher CoQ10 redox state [20]. However, in our analyses, the association between a higher redox state of CoQ10 and higher mortality risk appears to be independent of CRP levels, as associations held true after adjustment for CRP. Still, other markers implicated in inflammatory processes, such as TNF-α or IL-6, for which some studies have found reduced levels after CoQ10 supplementation [5] and others have not [4], might be implicated in this association. Unfortunately, TNF-α and IL-6 were not available for analysis in our cohort and should be investigated in future studies.

### 4.2. Strengths and Limitations

The comprehensive analysis of four markers of CoQ10 status, the medium-sized sample from the general population (*n* = 1333) and the long follow-up period (12.9 [12.4; 17.1] years) are considered strengths of our analyses. However, several limitations merit consideration. First, information on CoQ10 supplementation or other lifestyle and metabolic factors, such as dietary habits or renal function, which may influence both CoQ10 concentrations and mortality risk, were not available for our study sample. Second, despite our medium-sized sample, our study might not be sufficiently powered to detect smaller effect sizes, as detailed above. Third, even though we considered well-established risk factors in our multivariable adjusted analyses, we cannot entirely rule out residual confounding by other factors not available in our cohort. Fourth, as inherent to cross-sectional analyses, we could not take into account possible changes in CoQ10 status over the follow-up period, and no causality can be inferred from our observations. Fifth, the joint consideration of CoQ10 with other circulating antioxidant markers could corroborate our observations, yet these data were not available in our cohort. Lastly, since our sample comprises individuals from a specific region in Germany, our results may not be generalizable to other ethnicities.

## 5. Conclusions

In conclusion, we observed a higher redox state of circulating CoQ10 to be associated with a higher risk of all-cause mortality in a sample from the northern German general population. This observation might be indicative of detrimental long-term health effects of a lower antioxidant capacity of CoQ10 in the circulation and adds to the multi-faceted picture of CoQ10 in the biomedical literature. However, further studies that take into account other important antioxidants in addition to CoQ10 are needed to confirm our observation. Hence, clinical relevance of our findings for the general population remains to be determined.

## Figures and Tables

**Table 1:** Characterization of the overall study sample and stratified by vital status at the end of follow-up.

	Overall Sample (*n* = 1333)	Alive ^a^ (*n* = 1210)	Deceased ^a^ (*n* = 123)
Female sex, *n* (%)	801 (60.1%)	748 (61.8%)	53 (43.1%)
Age [years]	48.0 [37.7; 58.0]	47.0 [36.6; 56.0]	61.0 [50.8; 69.5]
Survival time ^a^ [years]	12.9 [12.4; 17.1]	13.0 [12.5; 17.2]	8.5 [4.8; 10.8]
Total Coenzyme Q10 [µmol/L]	0.82 [0.64; 1.03]	0.82 [0.64; 1.02]	0.90 [0.63; 1.09]
Ubiquinol [µmol/L]	0.71 [0.55; 0.89]	0.71 [0.55; 0.88]	0.76 [0.54; 0.94]
Ubiquinone [µmol/L]	0.11 [0.08; 0.13]	0.11 [0.08; 0.13]	0.12 [0.09; 0.14]
Coenzyme Q10 redox state ^b^ [%]	13.0 [11.8; 14.4]	12.9 [11.7; 14.3]	13.5 [12.4; 14.9]
Height [cm]	173 [167; 180]	172 [167; 180]	175 [166; 182]
Weight [kg]	85.0 [70.5; 103.5]	84.5 [70.0; 102.9]	93.9 [77.0; 119.7]
Body mass index [kg/m^2^]	27.5 [23.6; 34.8]	27.2 [23.5; 33.8]	30.5 [25.4; 40.5]
Systolic blood pressure [mmHg]	130 [120; 140]	130 [120; 140]	135 [130; 140]
Diastolic blood pressure [mmHg]	80 [75; 85]	80 [75; 85]	80 [80; 90]
C-reactive protein [mg/L]	1.7 [0.8; 4.5]	1.6 [0.8; 4.2]	3.0 [1.1; 7.2]
Total cholesterol [mmol/L]	4.7 [4.1; 5.3]	4.7 [4.1; 5.3]	4.7 [4.0; 5.3]
Glucose [mg/dL]	93 [87; 103]	93 [87; 101]	104 [93; 122]
Diabetes, *n* (%)	176 (13.2%)	131 (10.8%)	45 (36.6%)
Smoking habits			
Never smoked, *n* (%)	551 (41.3%)	510 (42.1%)	41 (33.3%)
Current smokers, *n* (%)	782 (58.7%)	700 (57.9%)	82 (66.7%)

Categorical variables are presented as *n* (%) and continuous variables as medians [quartile 1; quartile 3]. All values except for survival time and vital status are baseline characteristics collected at the baseline examination of the study sample. ^a^ At vital status assessment in 2024 (subsample A) and 2025 (subsample B and C). ^b^ Coenzyme Q10 redox state = percentage of ubiquinone in total Coenzyme Q10.

**Table 2:** Association between markers of Coenzyme Q10 status and all-cause mortality in *n* = 1333 individuals.

	Overall Sample (*n* = 1333)	Tertile 1 (*n* = 445)	Tertile 2 (*n* = 444)	Tertile 3 (*n* = 444)
Total Coenzyme Q10 [µmol/L]	0.82 [0.64; 1.03]	0.57 [0.48; 0.64]	0.82 [0.76; 0.88]	1.12 [1.03; 1.27]
Deceased, n (%)	123 (9.2%)	41 (9.2%)	31 (7.0%)	51 (11.5%)
	*Hazard Ratio and 95% Confidence Interval*			
Model 1	1.09 [0.92–1.29]	Reference	0.74 [0.46–1.17]	1.21 [0.80–1.83]
Model 2	0.98 [0.82–1.17]	Reference	0.56 [0.35–0.89]	0.92 [0.60–1.38]
Model 3	0.91 [0.73–1.14]	Reference	0.52 [0.32–0.85]	0.81 [0.49–1.33]
Model 4	0.92 [0.73–1.15]	Reference	0.51 [0.31–0.83]	0.81 [0.49–1.32]
				
Ubiquinol [µmol/L]	0.71 [0.55; 0.89]	0.50 [0.42; 0.55]	0.71 [0.66; 0.76]	0.97 [0.89; 1.12]
Deceased, n (%)	123 (9.2%)	41 (9.2%)	32 (7.2%)	50 (11.3%)
	*Hazard Ratio and 95% Confidence Interval*			
Model 1	1.07 [0.90–1.26]	Reference	0.77 [0.48–1.22]	1.19 [0.79–1.80]
Model 2	0.96 [0.80–1.15]	Reference	0.60 [0.37–0.95]	0.90 [0.59–1.36]
Model 3	0.89 [0.71–1.12]	Reference	0.57 [0.35– 0.92]	0.78 [0.48–1.29]
Model 4	0.90 [0.72–1.12]	Reference	0.55 [0.34–0.90]	0.78 [0.48–1.28]
				
Ubiquinone [µmol/L]	0.11 [0.08; 0.13]	0.07 [0.06; 0.08]	0.11 [0.10; 0.12]	0.15 [0.13; 0.17]
Deceased, n (%)	123 (9.2%)	33 (7.4%)	34 (7.7%)	56 (12.6%)
	*Hazard Ratio and 95% Confidence Interval*			
Model 1	1.23 [1.05–1.43]	Reference	1.03 [0.64–1.66]	1.70 [1.10–2.61]
Model 2	1.10 [0.93–1.31]	Reference	0.81 [0.50–1.31]	1.19 [0.77–1.83]
Model 3	1.07 [0.86–1.32]	Reference	0.77 [0.47–1.27]	1.12 [0.67–1.85]
Model 4	1.06 [0.86–1.32]	Reference	0.75 [0.45–1.23]	1.09 [0.66–1.81]
				
Coenzyme Q10 redox state ^a^ (%)	13.0 [11.8; 14.4]	11.2 [10.5; 11.8]	13.0 [12.6; 13.3]	15.0 [14.4; 16.4]
Deceased, n (%)	123 (9.2%)	23 (5.2%)	46 (10.4%)	54 (12.2%)
	*Hazard Ratio and 95% Confidence Interval*			
Model 1	1.28 [1.13–1.46]	Reference	2.13 [1.29–3.52]	2.57 [1.58–4.19]
Model 2	1.24 [1.07–1.43]	Reference	1.89 [1.14–3.13]	2.11 [1.28–3.46]
Model 3	1.19 [1.03–1.38]	Reference	1.81 [1.09–3.00]	1.95 [1.18–3.21]
Model 4	1.19 [1.03–1.37]	Reference	1.78 [1.07–2.96]	1.91 [1.15–3.15]
Model 5	1.18 [1.02–1.38]	Reference	1.77 [1.07–2.95]	1.88 [1.13–3.14]

Model 1: unadjusted; Model 2: adjusted for sex and age; Model 3: further adjusted for body mass index, smoking habits, systolic blood pressure, diabetes prevalence, and total cholesterol; Model 4: further adjusted for C-reactive protein; Model 5: further adjusted for total CoQ10. ^a^ Coenzyme Q10 redox state = percentage of ubiquinone in total Coenzyme Q10.

## Data Availability

The data are not publicly available due to legal and ethical restrictions. The data presented in this study are available on request through the application on the website https://portal.popgen.de/ (accessed on 25 January 2026).

## Supplementary Materials

The following supporting information can be downloaded at https://www.mdpi.com/article/10.3390/antiox15030343/s1, Figure S1: Flow Chart of eligibility of individuals for analyses according to the three subsamples comprising the analytical sample; Table S1: Characterization of the study sample according to the three subsamples included; Table S2: Association between markers of Coenzyme Q10 status and all-cause mortality in a sensitivity analysis excluding individuals who died within two years after baseline.
