# Supplementary material for: Higher Redox State of Coenzyme Q10 Is Associated with Higher Risk of All-Cause Mortality in a Sample from the Northern German General Population
Source: Antioxidants (Basel). 2026 Mar 9;15(3):343. doi: 10.3390/antiox15030343 (PMC13024566; doi:10.3390/antiox15030343)
Supplement: Supplementary file 1 [file antioxidants-15-00343-s001.zip › antioxidants-4150605-supplementary.pdf]

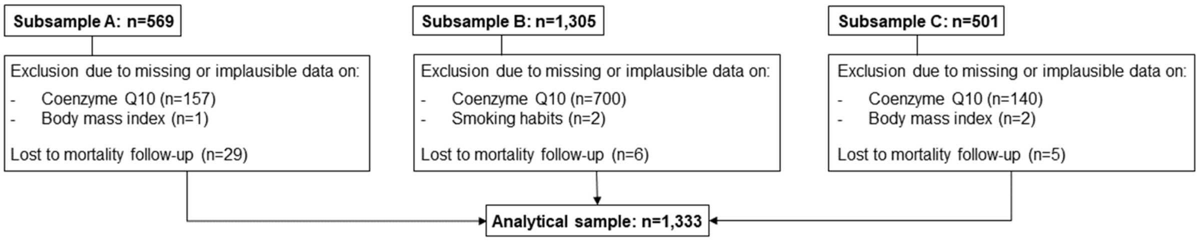

Figure S1: Flow Chart of eligibility of individuals for analyses according to the three subsamples comprising the analytical sample

Table S1: Characterization of the study sample according to the three subsamples included

	**Subsample A (n=382)**	**Subsample B (n=597)**	**Subsample C (n=354)**
**Female sex, n (%)**	179 (46.9%)	362 (60.6%)	260 (73.4%)
**Age [years]**	41 [31; 49]	54 [43; 66]	48 [40; 57]
**Deceased[a], n (%)**	12 (3.1%)	62 (10.4%)	49 (13.8%)
**Survival time [years]**	17.3 [17.2; 17.5]	12.5 [12.2; 12.8]	13.0 [12.3; 13.2]
**Total Coenzyme Q10 [µmol/L]**	0.89 [0.69; 1.12]	0.78 [0.61; 0.96]	0.80 [0.65; 0.99]
**Ubiquinol [µmol/L]**	0.78 [0.60; 0.98]	0.68 [0.52; 0.84]	0.70 [0.56; 0.87]
**Ubiquinone [µmol/L]**	0.11 [0.08; 0.14]	0.10 [0.08; 0.13]	0.11 [0.09; 0.13]
**Coenzyme Q10 redox state[b] [%]**	12.2 [10.9; 13.6]	13.2 [12.1; 14.4]	13.3 [12.2; 14.8]
**Height [cm]**	176 [169; 183]	172 [167; 180]	170 [165; 178]
**Weight [kg]**	79.0 [68.2; 90.0]	76.5 [65.8; 89.3]	122.0 [101.0; 144.0]
**BMI [kg/m²]**	25.4 [22.8; 28.1]	25.6 [22.7; 29.0]	42.0 [35.0; 48.4]
**Systolic blood pressure [mmHg]**	130 [120; 140]	130 [120; 135]	132 [130; 140]
**Diastolic blood pressure [mmHg]**	80 [70; 80]	80 [75; 80]	80 [80; 90]
**C-reactive protein [mg/L]**	1.0 [0.8; 2.5]	1.2 [0.8; 2.8]	5.6 [2.7; 11.0]
**Total cholesterol [mmol/L]**	5.0 [4.4; 5.6]	4.6 [4.0; 5.2]	4.5 [4.0; 5.1]
**Glucose [mg/dL]**	92 [86; 98]	93 [87; 100]	101 [90; 117]
**Diabetes, n (%)**	1 (0.3%)	53 (8.9%)	122 (34.5%)
**Smoking habits**			
**Never smoker, n (%)**	169 (44.2%)	251 (42.0%)	131 (37.0%)
**Ever smoker, n (%)**	213 (55.8%)	346 (58.0%)	223 (63.0%)

Categorical variables are presented as n (%) and continuous variables as median [interquartile range]. All values except for survival time and vital status are baseline characteristics collected at the baseline examination cycle of the study subsamples.
[a] At vital status assessment in 2024 (subsample A) and 2025 (subsample B and C)
[b] Coenzyme Q10 redox state = percentage of ubiquinone in total Coenzyme Q10

Table S2: Association between markers of Coenzyme Q10 status and all-cause mortality in a sensitivity analysis excluding individuals who died within two years after baseline

	Overall sample (n=1,325)	Tertile 1 (n=443)	Tertile 2 (n=442)	Tertile 3 (n=440)
**Total Coenzyme Q10 [µmol/L]**	0.82 [0.64; 1.03]	0.57 [0.48; 0.64]	0.82 [0.76; 0.88]	1.12 [1.03; 1.27]
**Deceased, n (%)**	115 (8.7%)	38 (8.6%)	30 (6.8%)	47 (10.7%)
	Hazard Ratio and 95% Confidence Interval			
**Model 1**	1.10 [0.93; 1.31]	Ref.	0.77 [0.48; 1.24]	1.2 0[0.78; 1.85]
**Model 2**	0.99 [0.82; 1.19]	Ref.	0.57 [0.35; 0.93]	0.90 [0.59; 1.39]
**Model 3**	0.91 [0.72; 1.14]	Ref.	0.52 [0.32; 0.87]	0.76 [0.45; 1.27]
**Model 4**	0.91 [0.72; 1.15]	Ref.	0.51 [0.31; 0.84]	0.76 [0.45; 1.27]
**Ubiquinol [µmol/L]**	0.71 [0.55; 0.89]	0.5 [0.42; 0.55]	0.71 [0.66; 0.76]	0.97 [0.89; 1.12]
**Deceased, n (%)**	115 (8.7%)	38 (8.6%)	30 (6.8%)	47 (10.7%)
	Hazard Ratio and 95% Confidence Interval			
**Model 1**	1.08 [0.90; 1.29]	Ref.	0.77 [0.48; 1.25]	1.20 [0.78; 1.84]
**Model 2**	0.97 [0.81; 1.17]	Ref.	0.59 [0.37; 0.96]	0.90 [0.59; 1.39]
**Model 3**	0.89 [0.70; 1.12]	Ref.	0.56 [0.34; 0.92]	0.76 [0.45; 1.27]
**Model 4**	0.89 [0.71; 1.13]	Ref.	0.54 [0.33; 0.89]	0.76 [0.45; 1.27]
**Ubiquinone [µmol/L]**	0.11 [0.08; 0.13]	0.07 [0.06; 0.08]	0.11 [0.1; 0.12]	0.15 [0.13; 0.17]
**Deceased, n (%)**	115 (8.7%)	30 (6.8%)	33 (7.4%)	52 (11.8%)
	Hazard Ratio and 95% Confidence Interval			
**Model 1**	1.23 [1.05; 1.45]	Ref.	1.10 [0.67; 1.80]	1.73 [1.10; 2.71]
**Model 2**	1.10 [0.92; 1.32]	Ref.	0.86 [0.53; 1.42]	1.20 [0.77; 1.89]
**Model 3**	1.05 [0.84; 1.32]	Ref.	0.81 [0.48; 1.35]	1.10 [0.65; 1.86]

**Model 4**	1.05 [0.84; 1.31]	Ref.	0.78 [0.46; 1.31]	1.07 [0.63; 1.82]
**Coenzyme Q10 redox state[a] (%)**	13.0 [11.8; 14.4]	11.2 [10.5; 11.8]	13.0 [12.6; 13.3]	15.0 [14.4; 16.3]
**Deceased, n (%)**	115 (8.7%)	21 (4.7%)	44 (10.0%)	50 (11.4%)
	Hazard Ratio and 95% Confidence Interval			
**Model 1**	1.28 [1.12; 1.46]	Ref.	2.25 [1.33; 3.78]	2.62 [1.57; 4.37]
**Model 2**	1.23 [1.06; 1.42]	Ref.	1.98 [1.17; 3.35]	2.14 [1.27; 3.58]
**Model 3**	1.19 [1.03; 1.39]	Ref.	1.92 [1.14; 3.25]	2.05 [1.22; 3.46]
**Model 4**	1.19 [1.02; 1.38]	Ref.	1.88 [1.11; 3.18]	2.00 [1.18; 3.39]
**Model 5**	1.18 [1.01; 1.39]	Ref.	1.87 [1.10; 3.17]	1.97 [1.16; 3.37]

Model 1: unadjusted; Model 2: adjusted for sex and age; Model 3: further adjusted for body mass index, smoking habits, systolic blood pressure, diabetes prevalence, and total cholesterol; Model 4: further adjusted for C-reactive protein; Model 5: further adjusted for total CoQ10

[a] Coenzyme Q10 redox state = percentage of ubiquinone in total Coenzyme Q10
